# Dotinurad: a novel selective urate reabsorption inhibitor as a future therapeutic option for hyperuricemia

**DOI:** 10.1007/s10157-019-01811-9

**Published:** 2019-11-21

**Authors:** Satoru Kuriyama

**Affiliations:** 1 Jikei University School of Medicine, Tokyo, Japan; 2 Nephrology and Hypertension Research Unit, Miho Clinic, 1-6-4, Osaki, Shinagawa-ku, Tokyo, 141-0032 Japan

**Keywords:** Dotinurad, URAT-1, Selective urate reabsorption inhibitor (SURI), Chronic kidney disease, Gout, Hyperuricemia

## Abstract

Gout is a chronic inflammatory disease caused by precipitation of urate crystals in the joints, kidneys, and urinary tract. Independent of urate deposition disorders, recent studies have shown a positive association between circulating uric acid (UA) levels and cardiovascular (CV) diseases. These results indicate that UA is a precipitating factor of both gout and the progression of CV diseases, including hypertension and/or chronic kidney disease (CKD). A large body of evidence has shown that UA-lowering therapies are effective in preventing the progression of hypertension/CKD and that a causal relationship exists between serum UA level and CV diseases. Despite the urgent need for effective UA-lowering drugs that can be used to obtain better therapeutic outcomes and prognosis, only few drugs have been developed in the past decades. Recently, febuxostat and topiroxostat, which are xanthine oxidoreductase inhibitors, were developed and used in clinical practice. Of note, after the approval of lesinurad, which is a urate transporter-1 (URAT-1) inhibitor, in the United States in 2015, dotinurad (Fig. 1), a novel promising drug with selective UA reabsorption inhibitory property, was recently developed in Japan in 2018. Dotinurad is indicated for patients with hyperuricemia/gout as most patients with hyperuricemia are classified into “underexcretion type”, which requires the inhibition of URAT-1 to excrete excess UA via the kidney. Focusing on dotinurad, the present study highlighted the multifaceted preliminary new trials that assessed for drug efficacy and safety, pharmacokinetics (PK) according to age and gender, the presence or absence of liver and kidney disorders, drug interactions with NSAID, and non-inferiority of dotinurad to either febuxostat or benzbromarone. A series of studies included in this supplemental review indicate that dotinurad reduces serum UA levels, and its efficacy and safety are similar to those of other UA-lowering agents currently used even in hyperuricemic patients with various clinical conditions. Moreover, two exploratory studies with a small sample size were conducted to compare PK parameters between patients with overproduction- and underexcretion-type hyperuricemia, and results showed that the effects of UA-lowering agents were comparable between the two subtype groups.

**Fig. 1:**
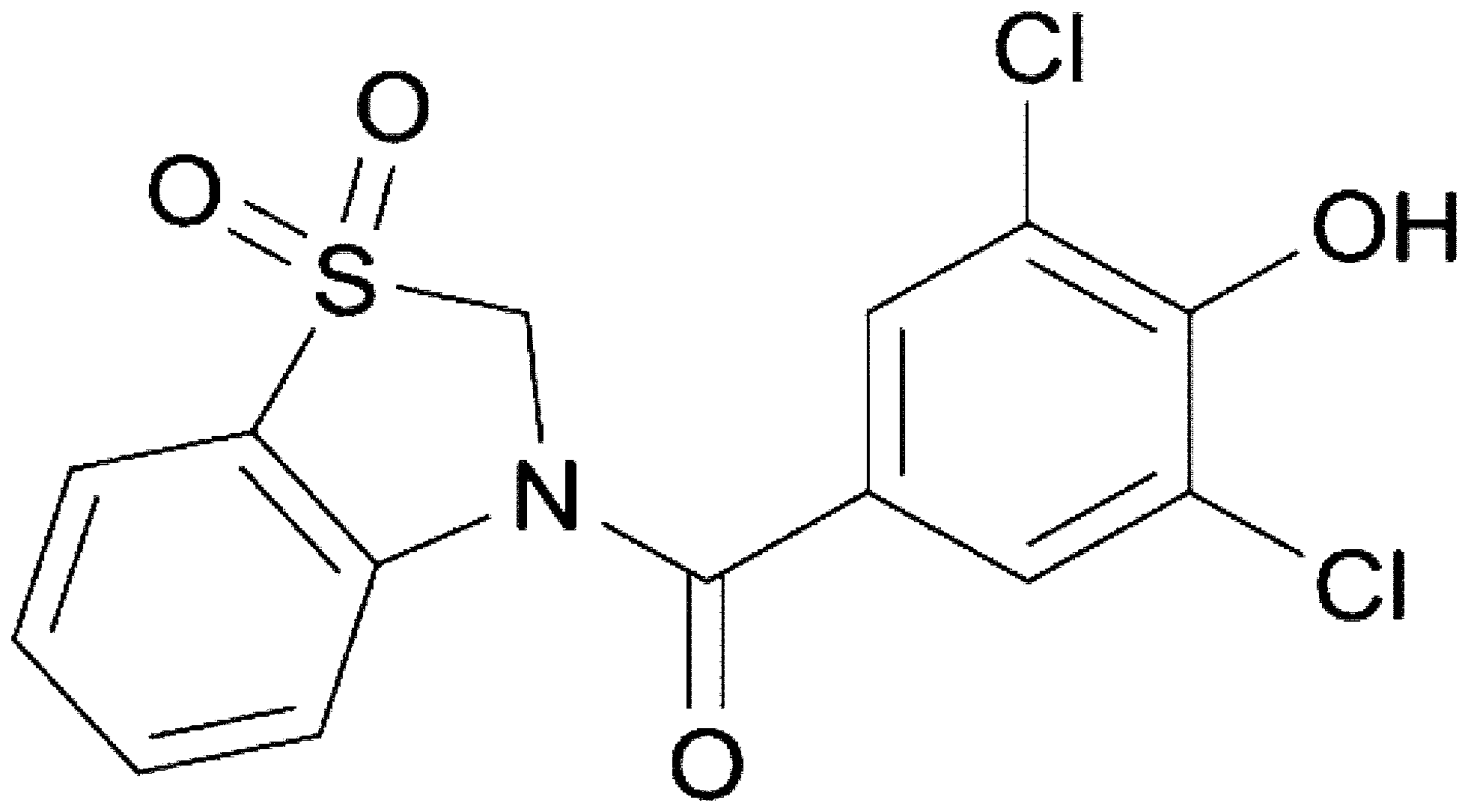
Chemical structural formula of dotinurad

Chemical structural formula of dotinurad

## Introduction

The number of patients with hyperuricemia has continuously increased in recent years due to the increased occurrence of metabolic syndrome. Hyperuricemia causes gouty arthritis, gouty kidney, and urolithiasis. Evidence has shown that hyperuricemia is also associated with hypertension [1–5], chronic kidney disease (CKD) [6, 7] and end-stage renal disease [8]. This evidence leads to the question whether hyperuricemia plays a pivotal role in the pathogenesis of various cardiovascular (CV) diseases. Weather UA per se is a factor that exacerbates the progression of CV disease is a matter for debate. Studies that investigate whether UA-lowering therapy is effective for cardiorenal protection partly answers the question because they provides an insight about the causal relationship between increased UA levels and CV diseases. Indeed, a substantial number of reviews and meta-analysis that were recently conducted has shown that UA-lowering therapies retard the progression of cardiorenal disease, thereby leading to a better prognosis [9–14].

Regarding the pathogenesis of CKD, UA-induced renal arteriopathy can explain the primary mechanism by which hyperuricemia causes impairment in renal function. UA results in a higher risk of hyalinosis and higher-grade wall thickening in patients with biopsy-proven CKD [15]. In addition, a positive correlation was observed between serum UA levels and intra-glomerular pressure, but not between serum UA levels and estimated glomerular filtration rate (eGFR). Thus, individuals with hyperuricemia are highly at risk for a progressive decline in renal function [16]. Feig et al. [17, 18] have proposed that elevated UA levels can lead to vasoconstriction-mediated hypertension at an early stage, followed by volume-dependent hypertension with subsequent renal damage at a later stage. This hypothesis is endorsed later by the observations that UA induces renal ischemia which activates renal RAS [16], and acts on epithelial Na channel in the tubules to reabsorp Na which leads to volume expansion [19].

In most individuals, UA levels are genetically determined. Clinical studies of patients with hyperuricemia indicate that the contribution of heritability to hyperuricemia is approximately 70% or higher [20, 21]. This result indicates that life-style modifications, such as dietary interventions or alcohol restriction, are not always effective in reducing UA levels to the physiological normal range. Thus, pharmacological interventions are essential in effectively decreasing UA levels, which leads to better treatment outcomes and prognosis.

Allopurinol, a xanthine oxidoreductase (XOR) inhibitor developed in the 1960s, and benzbromarone (BZB), a urate transporter 1 (URAT-1) inhibitor developed in the 1970s, are long-term drugs widely utilized for hyperuricemia/gout. Since then, only few drugs with UA-lowering potency have been available until quite recently. Febuxostat and topiroxostat, which are XOR inhibitors and alternative drugs to allopurinol, have been developed and widely used within the last 10 years.

Regarding drugs with URAT-1 inhibitory property, the Food and Drug Administration (FDA) approved the use of lesinurad in the United States in 2015. Subsequently, dotinurad, a novel promising drug with selective urate reabsorption inhibitor (SURI) property, was recently developed in Japan in 2018.

Generally, hyperuricemia is classified into the overproduction type and underexcretion type based on the amount of renal UA excretion. Because the prevalence of the underexcretion type is evidently higher (approximately 60% or higher) than that of the overproduction type in patients with Japanese ethnic extraction [22], the importance of choosing drugs with SURI property will be inevitably and naturally expanding in the future.

Assuming that dotinurad will soon be available in the market, the present review summarized the basic and clinical information obtained from the 11 preliminary clinical trials that assessed the following: (1) efficacy and safety, (2) pharmacokinetics (PK), (3) evaluation results about the non-inferiority of dotinurad to other UA-lowering agents, (4) effects of age, gender, and liver and kidney diseases, (5) factors associated with overproduction- and underexcretion-type hyperuricemia, and (6) drug interaction with NSAIDs.

## Efficacy and safety of dotinurad (phase 2a, 2b and phase 3 studies)

Early- and late-phase clinical trials (phase 2a and 2b studies) were carried out to determine the optimal dose of dotinurad, and phase 3 study was performed to assess the long-term effect of such drug [23–25] (Table 1).

**Table 1:** Clinical trials of dotinurad

References	Clinical trails gov ID	Study objectives	Subjects or patients	Dotinurad dose (day)	Dosing period
[23]	NCT02344862	Dose response, optimal dose and safety (phase 2a)	Hyperuricemia	0.25 → 0.5 → 1,2,4 mg placebo	8 weeks
[24]	NCT02416167	Dose response, optimal dose and safety (phase 2b)	Hyperuricemia	0.25 → 0.5 → 0.5,1,2,4 mg placebo	12 weeks
[25]	NCT03006445	Long-term efficacy and safety	Hyperuricemia	0.5 → 1→2 mg 0.5 → 1→2 → 4 mg	34 or 58 weeks
[26]	NCT02344875	PK, PD, and safety in elder subjects	Elderly	1 mg	Single dose
[27]	NCT02347046	PK, PD, and safety in patients with CKD	CKD Healthy	1 mg	Single dose
[28]	NCT03306667	PK, PD, and safety in patients with liver damage	Liver disease Healthy	4 mg	Single dose
[30]	NCT03100318	Non-inferiority test to benzbromarone and evaluation of safety	Hyperuricemia	0.5 → 1→2 mg benzbromarone 25 → 50 → 50 mg	14 weeks
[31]	NCT03372200	Non-inferiority test to febuxostat and evaluation of safety	Hyperuricemia	0.5 → 1→2 mg febuxostat 10 → 20 → 40 mg	14 weeks
[32]	NCT03350386	PK and safety of oxaprozin in combination (Drug interaction)	Healthy	4 mg → oxaprozin 600 mg → 4 mg + oxaprozin 600 mg	Single dose 6 days
[33]	NCT02837198	PD, PK, and safety in patient groups classified into G1 and G2	G1: overproduction type, G2: underexcretion type	1 mg → 1 mg + topiroxostat 80 mg	7 days
[34]	NCT03375632	PD and safety in patient groups classified into G1 and G2	G1 and G2	0.5 → 1→2 → 4 mg	14 weeks

*PK* Pharmacokinetics, *PD* Pharmacodynamics, *UA* Uric acid, *CKD* Chronic kidney disease

Phase 2a and 2b trials were randomized, multicenter, double-blind, placebo-controlled, parallel-group, and dose-escalation studies. A total of 80 patients were recruited in the phase 2a trial, which revealed that serum UA levels substantially decreased when dotinurad was administered at a dose of 1, 2, and 4 mg for 8 weeks in a dose-dependent manner [23]. Similarly, more than 200 patients were recruited in the confirmatory phase 2b study with an observation period of 12 weeks, and such study validated that serum UA levels decreased when dotinurad was administered at a dose of 0.5, 1, 2, and 4 mg in a dose-dependent manner [24].

In addition, the phase 3 trial, a long-term observation study, was independently planned and carried out. More than 300 patients were recruited in this multicenter, open-label, dose-escalation study performed at 26 medical institutions to evaluate the long-term efficacy and safety of dotinurad. The results indicated that the decrease in UA levels was significant even at an early time (2 weeks), and the efficacy was continuously observed throughout an observation period of 58 weeks [25]. The serum UA level decreased by about 20% at a dose of 0.5 mg after week 2, and the level was persistently low at 40–60% throughout the study period. The achievement rate of a serum UA level at less than 6 mg/dL was about 80–90% at a maintenance dose of 2 mg after week 10, and the effect remained stable throughout the observation period. A slight increase in γ-GTP was observed after 34 weeks of using dotinurad 2 and 4 mg. However, such increase was transient, and no additional abnormality was observed until week 58. Findings showed that < 1% of patients had new onset of gouty arthritis from week 34 to week 58 and that the achievement rate of a serum UA level < 6.0 mg/dL was high at approximately 90%, which indicates that dotinurad can inhibit the development of new-onset gouty arthritis.

Based on the findings obtained in the trial, dotinurad is effective in reducing serum UA levels during the long-term observation period.

## Pharmacokinetics of dotinurad

In general, PK should be analyzed in various clinical conditions as UA metabolism might be influenced by age and gender, and renal and liver clearances are the two major factors that determine PK parameters. Thus, preliminary PK trials about dotinurad were performed in multiple clinical settings [26–28] (Table 1). A series of measurements revealed that *T*_1/2_ was approximately 10 h, regardless of the dosage, age, and gender [26], and that only mild-to-moderate kidney and liver dysfunctions were found [27, 28]. Based on these observations and the phase 2 and 3 trial results, dotinurad can be administered at a dose between 0.5 and 4 mg to ensure and provide optimal UA-lowering effects, which can be maintained for a long period of time. Moreover, dotinurad can be administered once a day.

## Non-inferiority trials of dotinurad to benzbromarone and febuxostat

In general, clinical trials that assess whether dotinurad is not inferior to other currently available UA-lowering agents may be essential. Non-inferiority clinical trials have been recommended to establish the efficacy of newly developed drugs. For instance, in the CARES trial, the FDA required that febuxostat (FBX) should be compared with placebo and allopurinol to assess the inferiority of FBX to other UA-lowering drugs [29].

In phase 3 trial, Hosoya et al. have investigated the non-inferiority of dotinurad to either BZB or FBX in two separate trials [30, 31] (Table 1). In a randomized, multicenter, double-blind, dose-escalation, double-dummy, BZB-controlled study, a total of 201 patients were randomly assigned to treatments with either dotinurad 2 mg or BZB 50 mg once a day for 14 weeks in a dose-escalation manner to compare the efficacy and safety of the two drugs [30]. In another set of non-inferiority trial of FBX versus dotinurad, a total of 203 patients were randomly assigned to treatment with either dotinurad 2 mg or FBX 40 mg once a day for 14 weeks in a dose-escalation manner [31].

These two studies clearly showed that dotinurad is not inferior to either FBX or BZB in lowering serum UA levels. In addition, no notable adverse events were observed in the dotinurad-, FBX-, and BZB-treated groups.

## Drug interaction with NSAIDs

The pharmacokinetic interactions of the combined dosing of dotinurad and NSAIDs will be clinically crucial as NSAIDs are frequently administered in combination with UA-lowering agents in patients who present with intractable pain due to gouty arthropathy. In vitro experiments indicated that oxaprozin, a non-steroidal anti-inflammatory drug, may be the most apprehensive candidate among the potential co-administered drugs (such as NSAIDs, XOIs, anti-hypertensive drugs, anti-hyperlipidemic drugs, anti-diabetic drugs, and others), which can interact with dotinurad by interfering the glucuronidation conjugate process and decreasing the plasma protein binding rate of dotinurad. In a study that recruited healthy participants, the interaction between oxaprozin and dotinurad was investigated by measuring the blood concentration of dotinurad and UA levels. Although the T_1/2_ and AUC_(0-Inf)_ of dotinurad increased slightly when dotinurad was co-administered with oxaprozin compared to dotinurad alone, the results were still within the acceptable clinical range [32] (Table 1). If one can extrapolate this result to other NSAIDs that are less harmful than oxaprozin, the combined use of dotinurad and the commonly used NSAIDs, such as naproxen, pranoprofen, and/or indometacin, may result in less problems.

## Dotinurad as a future therapeutic option for hyperuricemia/gout

As previously mentioned, the predominance of the underexcretion type over the overproduction type highlights the emerging attention for drugs with SURI property. In daily clinical practice, physicians are not always meticulous about making a differential diagnosis of the subtype of hyperuricemia because the measurement of urinary UA excretion is time- and effort-consuming. Thus, even if dotinurad was arbitrarily chosen for a patient whose clinical subtype was unknown, one might still expect a secure UA-lowering effect. To address such issue, the effects of the clinical subtypes of hyperuricemia were the focus of two separate trials [33, 34] (Table 1). Namely, Hosoya et al. and Okui et al. have conducted clinical studies that explored whether dotinurad has different effects between patients with overproduction- and underexcretion-type hyperuricemia in terms of the amount of urinary UA excretion and UA reduction rate by screening inpatients based on two categories. The study indicated that these parameters and the PK parameters were comparable between the two groups [33]. Then, the study was extended to the outpatient setting to confirm whether the abovementioned findings obtained from inpatients are accurate. This outpatient study showed reproducibly that the UA-lowering rates and/or the rate of urinary UA excretion was comparable between patients who overproduce and underexcrete UA. In both groups, urinary UA excretion transiently increased and then returned to the steady-state level, thereby maintaining a slightly increased level [34].

However, one must be alert to the potential fear of enhancing UA stone formation induced by uricosuric agents. Although the prevalence of urinary stones of UA origin is approximately 1–3% [35] among all patients who were diagnosed with urolithiasis, whether the use of dotinurad may not directly lead to an increase in UA stone formation is not known. Since drugs with SURI property causes uricosuria, the potential risk for UA stone formation should be carefully monitored in the future observation.

Finally, the side effects of lesinurad, which is an URAT-1 inhibitor that was recently made available in the market, has gained clinical attention. In terms of the side effects of such drug, the main concern is the increase in serum creatinine concentration [36]. Because hyperuricemia/gout is a chronic condition and its therapy must continue for an extended period, cautious observation of any potential side effects, including renal damage, is also crucial in future treatments with dotinurad.

I feel that dotinurad may practically be given to both subtypes of hyperuricemia/gout patients assuming that the PK parameters are almost comparable between the two subtypes and that the adherence rate of measuring urinary UA excretion rate in the outpatient clinic is quite low. Certainly, it is too early to evaluate the intrinsic advantages of dotinurad. However, drugs with SURI property can be used to achieve the purpose of effectively lowering UA levels, thereby leading to better treatment outcomes in patients with hyperuricemia/gout.

## Conclusions

Dotinurad is effective in lowering serum UA levels, and its efficacy and safety are similar to those of other UA-lowering drugs in patients with hyperuricemia/gout.
